# Kawasaki Disease and Allergic Diseases

**DOI:** 10.3389/fped.2020.614386

**Published:** 2021-01-07

**Authors:** Po-Yu Huang, Ying-Hsien Huang, Mindy Ming-Huey Guo, Ling-Sai Chang, Ho-Chang Kuo

**Affiliations:** ^1^Department of Traditional Chinese Medicine, Kaohsiung Chang Gung Memorial Hospital and Chang Gung University College of Medicine, Kaohsiung, Taiwan; ^2^Department of Pediatrics, Kawasaki Disease Center, College of Medicine, Kaohsiung Chang Gung Memorial Hospital and Chang Gung University, Kaohsiung, Taiwan; ^3^Graduate Institute of Clinical Medical Sciences, College of Medicine, Chang Gung University, Kaohsiung, Taiwan; ^4^Department of Respiratory Therapy, Kaohsiung Chang Gung Memorial Hospital, Kaohsiung, Taiwan

**Keywords:** allergic diseases, allergic rhinitis, asthma, atopic dermatitis, Kawasaki disease

## Abstract

**Background:** Kawasaki disease (KD) is an inflammatory disorder with an unknown etiology. It is the leading cause of acquired heart disease, which leads to coronary vasculitis among children. Studies of frequent manifestation of allergic diseases in children with KD have been the subject of mounting clinical interest. However, evidence supporting the association between KD and allergies has yet to be systematically reviewed.

**Methods:** In this article, we reviewed current literature regarding the association between KD and allergic diseases. References for this review were identified through searches of PubMed, Cochrane, and Embase through the end of August 2020.

**Results:** The results of the analyses of immune repertoire, clinical, and epidemiological studies have indicated some of the characteristics of infectious disease for KD. Although some allergic disorders, such as asthma, may be exacerbated by viral infections, allergies are typically caused by an allergen that triggers an immune response, with the potential involvement of type 2 inflammation and immune disturbances leading to tissue remodeling in genetically susceptible hosts. The effect of intravenous immunoglobulin is multi-faceted and results in a decrease in activating Fc gamma receptor IIA and an increase in anti-inflammatory eosinophils. The findings from this review demonstrate that children who have suffered from KD are more likely to have allergic rhinitis than the general population and their siblings, a condition that lasts until the age of 17. When followed up as teenagers and adults, children with KD are more likely to develop urticaria.

**Conclusions:** This review supports that allergic diseases, such as allergic rhinitis, have been demonstrated to increase following KD. Therefore, the importance of allergic diseases in patients with KD should be emphasized in long-term care. Interventions that include strategies for managing allergies in children with KD would be beneficial.

## Introduction

**Figure 1:**
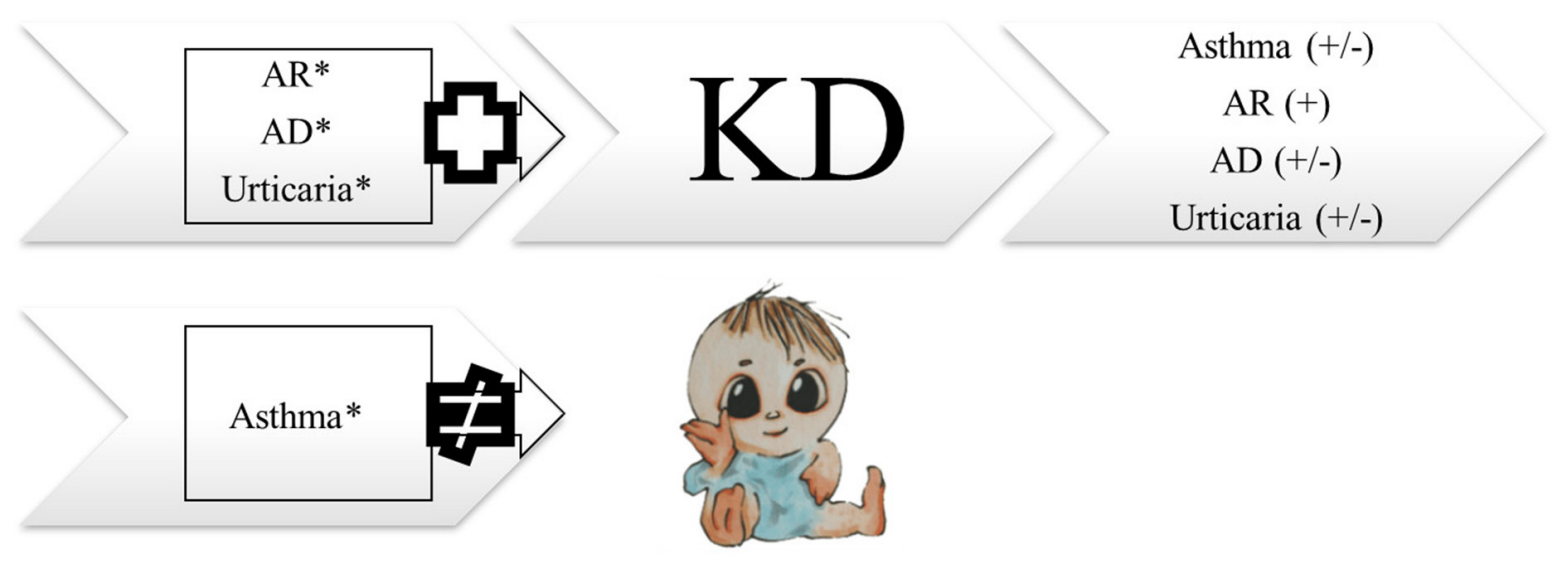
The association between Kawasaki disease and allergic diseases. AD, atopic dermatitis; AR, allergic rhinitis; KD, Kawasaki disease. *derived from Wei et al. (28).

**Figure 2:**
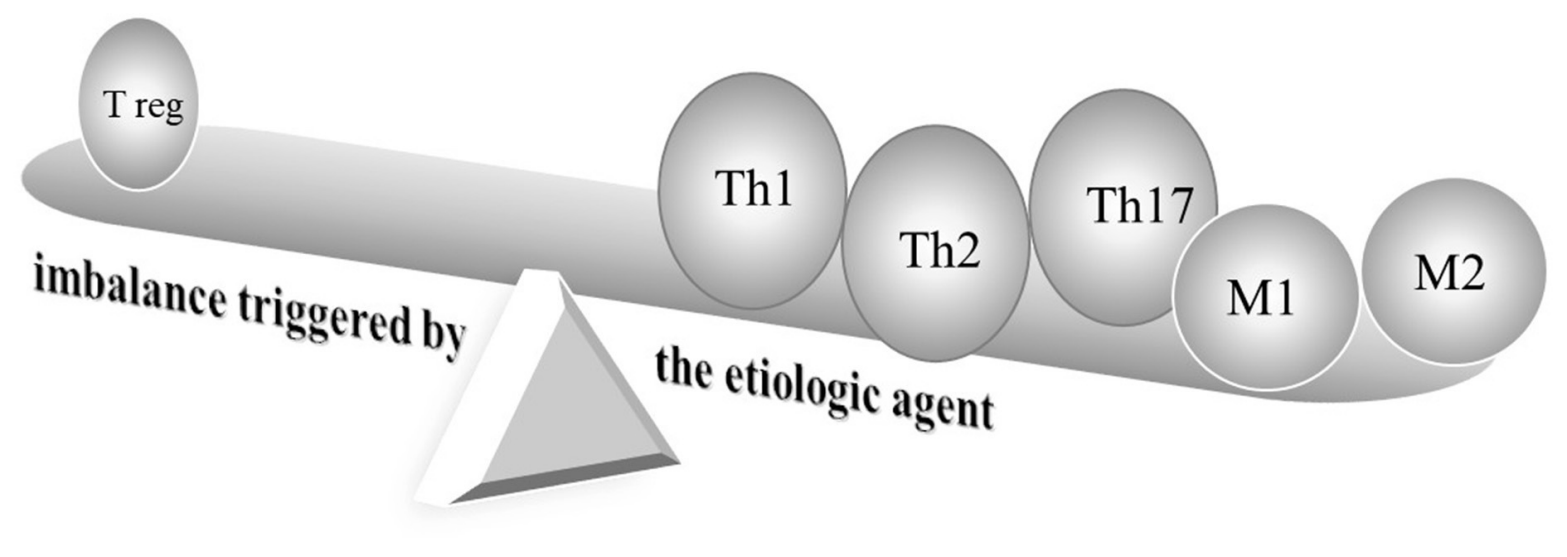
Schematic diagram of immune responses in Kawasaki disease. Cutting-edge research has indicated the deterioration of T helper type (Th)1, Th2, Th17, and regulatory T cells, as well as activated and alternatively activated macrophages in KD. M, macrophage; reg, regulatory; Th, T-helper.

Kawasaki disease (KD) is a major acquired vasculitis that occurs in childhood and has had increasing incidence worldwide. Accumulating evidence has suggested that KD and allergic diseases may be reciprocally linked (Figure 1). Although the etiology of KD remains unclear and diverse, a T-helper (Th)1/Th2 imbalance is triggered by the etiologic agent (Figure 2) (1, 2). While KD is a complex, multifaceted illness, an underlying allergic immune response may play a role in KD severity. An association between KD and allergy has long been touted, and KD has been found to be a contributor to the development of allergic diseases.

As increasing numbers of KD patients are reaching adulthood, more information is becoming available regarding the long-term consequences 50 years after KD was first described (3). Reports of cognitive development and overall psychosocial performance have provided reassurance that KD does not affect developmental milestones or cognitive impairment (4, 5). Lin et al. (6) observed various and more neurodevelopmental disorders in a study following 612 KD patients. Although many aspects of long-term clinical follow-up of previous KD and lifetime approaches remain unknown, long-term sequelae, particularly coronary aneurysm, continue to be a major concern in the management of this disease (7). One hundred KD patients with or without coronary artery dilatation showed more subclinical abnormalities of left ventricular function than healthy controls in children more than 7 years after KD (8). Autoantibodies, a target for long-term assessment, were found to last for a long time in children with KD (9). Lipid profile abnormalities in KD were found to persist for 1 year in a cohort study with 27 children (10). In addition to cardiovascular sequelae, KD has been demonstrated to have other long-term effects resulting in common health problems, such as allergy diseases and impaired immunity (11, 12). In this review article, the current understanding of the link between allergies and KD are discussed (Figure 1).

## Search Strategy and Selection Criteria

References for this review were identified through searches of PubMed with the search terms “Kawasaki disease^*^” AND (allerg^*^ OR atop^*^ OR asthma OR rhinitis OR dermatitis OR eczema), Cochrane with the search terms “Kawasaki disease,” Embase with the search terms “Kawasaki disease and eczema,” “Kawasaki disease and dermatitis,” “Kawasaki disease and rhinitis,” or “Kawasaki disease and asthma” until the end of August 2020. We selected references that had already been published and were drafted in English.

## Possible Association With Increased Allergic Diseases Among Post-KD Patients

Several population-based studies have enhanced the understanding of the interaction between KD and allergic diseases. Kuo et al.'s (13) study clearly demonstrated an association between KD and the subsequent 1.51 and 1.30-fold risk of asthma (Table 1) and allergic rhinitis (Table 2), respectively, using 253 KD patients who were 5 years of age or younger during the 5-year follow-up period. In many cases, children with KD are also diagnosed with and treated for allergic diseases after KD. In Taiwan, the incidence rate of allergy in patients with KD is 184.66 per 1,000 person-years during the five-year follow-up period, with varying symptoms. Of those, 62.19 per 1,000 person-years in patients with KD have asthma, and 95.45 per 1,000 person-years have allergic rhinitis. Another population-based cohort study in Taiwan on the correlation of KD and allergic diseases in school-age children demonstrated a significantly greater subsequent risk of asthma (odds ratio, 1.16) and allergic rhinitis (odds ratio, 1.30) in the KD group (*n* = 7,072) than in the non-KD controls (*n* = 27,265) (14). Meanwhile, Taiwanese schoolchildren who had suffered from KD did not experience an increase in the occurrence of atopic dermatitis (Table 3) or urticaria (Table 4) compared to the paired controls (14). Hwang et al. (15) observed an increased risk of the concomitant occurrence of asthma and allergic rhinitis in children with a prior diagnosis of KD younger than 5 years of age based on an analysis of a large population-based database. Over 60% of the KD patients developed allergic diseases after the KD diagnosis. However, no significant difference was observed in the risk of atopic dermatitis between the KD group and the control group, and KD tended to occur before chronic urticaria onset before the age of 20 years old (Figure 1) (16). Woon's nationwide, population-based, longitudinal study of 360 preschool KD children ≤ 6 years old for a 5-year follow-up demonstrated an association between KD and the risk of atopic dermatitis by ~1.25 times (*p* = 0.04) (17). To study the relationship between KD and atopic dermatitis, a previous case-control telephone survey by Brosius et al. (18) also showed a nine-time increased prevalence of atopic dermatitis in KD.

**Table 1:** Effect of Kawasaki disease on asthma.

**Study**	**Study design**	**Population**	***N* number of KD patients**	**Age of KD patients (y)**	**Control**	**Follow-up period (y)**	**Adjusted Hazard ratio**	**CI**
(13)	Population-based	Taiwan	253	5 years of age or younger	Non-KD	5-years	1.51	1.17–1.95
(14)	Population-based	Taiwan	7,072	0–5 years	Non-KD	Till 6–13 years old	1.03 (adjusted OR)	0.93–1.13
(23)	Cross-sectional study	Israel	144		General population	17 years old at evaluation	2.919 (OR)	1.682–5.067
(24)	Cross-sectional study	Singapore	93	1.17	Well-sibling controls	7.08 years old at evaluation	2.56 (adjusted OR)	0.80–8.23
**Concomitant**								
(15)	Population-based	Taiwan	200	Younger than the age of 5 years	Non-KD		1.19 (adjusted OR)	0.80–1.78

*CI, confidence interval; KD, Kawasaki disease; OR, odds ratio; y, years*.

**Table 2:** Effect of Kawasaki disease on allergic rhinitis.

**Study**	**Study design**	**Population**	***N* number of KD patients**	**Age of KD patients (y)**	**Control**	**Follow-up period (y)**	**Adjusted Hazard ratio**	**CI**
(13)	Population-based	Taiwan	253	5 years of age or younger	Non-KD	5-year	1.30	1.04–1.62
(14)	Population-based	Taiwan	7,072	0–5 years	Non-KD	Till 6–13 years old	1.28 (adjusted OR)	1.20–1.37
(19)	Case-control	Japan	1,165	KD between the ages of 1 and 5 years	Non-KD		1.69 (relative risk)	
	Questionnaire							
	Survey							
(23)	Cross-sectional study	Israel	144		General population	17 years old at evaluation	2.634 (OR)	1.686–4.114
(24)	Cross-sectional study	Singapore	93	1.17	Well-sibling controls	7.08 years old at evaluation	2.90 (adjusted OR)	1.27–6.60
**Concomitant**								
(15)	Population-based	Taiwan	200	Younger than the age of 5 years	Non-KD		1.31 (adjusted OR)	0.88–1.94

*CI, confidence interval; KD, Kawasaki disease; OR, odds ratio; y, years*.

**Table 3:** Effect of Kawasaki disease on atopic dermatitis.

**Study**	**Study design**	**Population**	***N* number of KD patients**	**Age of KD patients (y)**	**Control**	**Follow-up period (y)**	**Adjusted Hazard ratio**	**CI**
(14)	Population-based	Taiwan	7,072	0–5 years	Non-KD	Till 6–13 years old	0.95 (adjusted OR)	0.82–1.10
(17)	Population-based	Taiwan	360	0–6 years	Non-KD	5 years	1.25	1.01–1.54
(18)	Case-control telephone survey	United States	83		Innocent heart murmurs		9	1.6–49.4
(19)	Case-control	Japan	1,165	KD between the ages of 1 and 5 years	Non-KD		1.66 (relative risk)	
	Questionnaire							
	Survey							
(24)	Cross-sectional study	Singapore	93	1.17	Well-sibling controls	7.08 years old at evaluation	1.30 (adjusted OR)	0.52–3.23
**Concomitant**								
(15)	Population-based	Taiwan	200	Male subjects with KD between 1 and 5 years of age	non-KD		3.02 (OR)	1.22–7.50

*CI, confidence interval; KD, Kawasaki disease; OR, odds ratio; y, years*.

**Table 4:** Effect of Kawasaki disease on urticarial.

**Study**	**Study design**	**Population**	***N* number of KD patients**	**Control**	**Follow-up period (y)**	**OR**	**CI**
(14)	Population-based	Taiwan	7,072	Non-KD	Till 6–13 years old	1.04 (adjusted OR)	0.93–1.15
(16)	Population-based	Taiwan	9	Non-urticaria	37.7 at evaluation	2.76 (adjusted OR)	1.15–6.63
(23)	Cross-sectional study	Israel	144	General population	17 years old at evaluation	7.706	3.156–18.813

*CI, confidence interval; KD, Kawasaki disease; OR, odds ratio; y, years*.

Collecting a parental questionnaire of KD patients revealed a higher incidence of both atopic dermatitis and allergic rhinitis in KD patients without a family history vs. the control children (19). In the KD patients with atopic family history, the incidence of allergic conjunctivitis and drug allergy also differed significantly between the KD and control subjects. The incidence of asthma did not differ significantly between the KD patients and controls, perhaps because the questionnaire response rate from KD patients was much lower than that of the control group. Another possible reason may have been that the environmental factors of the KD children in this study showed fewer households with pets or cigarette smoking. The incidence of food allergy defined as reproducible allergic symptoms by a specific food in KD children did not differ from controls (19). Interleukin (IL)-10 enhancing immunoglobulin (Ig)E-mediated mast cell activation was essential for developing food allergies in a murine model (20, 21). Lee et al. (22) found significantly elevated IL-10 in patients with KD. The arguments regarding food allergy in children with KD need to be confirmed by further studies.

A large-scale cross-sectional study involving teenagers aged 16–20 years with a medical history of KD both with and without cardiac manifestation suggested that KD is a risk factor for asthma, allergic rhinitis, and chronic urticaria. This association increases with the cardiac manifestation of KD (23). Another questionnaire design in a cross-sectional study aimed to evaluate the possible tendency toward allergic diseases in KD (24). The unaffected siblings of KD probands were less likely to develop allergic rhinitis compared with their KD siblings. KD children without coronary artery lesions (CAL) have an intensified form of allergic rhinitis and any other allergies when compared with their sibling controls, consistent with prior observations of lower Th2 cytokines in KD patients with CAL, suggesting that Th2 plays a protective role in KD (25). Whether CAL was protective of subsequent allergic diseases or not was not entirely clear based on this conflicting evidence. Furthermore, children who were diagnosed with KD older than 1 year old had more asthma and allergies when compared with their sibling controls, indicating an immune tolerance to allergens at a young age.

Altogether, a majority of findings from the studies was classified as having a higher risk of allergic diseases, especially allergic rhinitis in children with KD (Table 2). Approaches that include strategies for following up allergies and anti-allergic therapy would be beneficial in children with KD.

## Concurrent Allergic Diseases During and Prior to the Onset of Kawasaki Disease

A genetic predisposition to allergies may be associated with a susceptibility to KD immunologically programmed to overreact to such irritants as infection or antigen entry from skin and mucous membranes (19). A family history of allergies was significantly more common in children with KD than in controls (19). According to a report by Burns et al. (26), their family-based genotyping study of KD patients suggested that genetic variation in the IL-4 (-589) gene played an important role in KD pathogenesis and disease susceptibility. Although no changes in expression of the IL-4 receptor have been associated with the minor allele of rs563535954, it has played a role in Japanese KD patients with poor response to intravenous immunoglobulin (IVIG) (27).

Children who have previously suffered from combined allergic diseases (atopic dermatitis, allergic rhinitis, and urticaria) are at an increased risk of developing KD diagnosed at a mean of 2.83 years later in life (Figure 1) (28). Furthermore, Wei et al. (28) found an increased risk of KD in children who had sought medical care for allergic diseases more than twice a year compared to children with fewer medical visits. Gender-stratified analysis showed a significantly increased cumulative risk of KD in the follow-up period in male children with atopic dermatitis and in both genders with regard to allergic rhinitis and urticaria patients. However, in this study, increased risk of KD was not noted in children who had previously had asthma or allergic conjunctivitis (Figure 1, Table 5). Furthermore, this population-based case-control study of 2,748 patients with KD demonstrated that concomitant allergic diseases, such as acute conjunctivitis, were significantly higher in the KD group compared with the non-KD group.

**Table 5:** Allergic diseases and subsequent risk of Kawasaki disease.

**Allergic diseases**	**Study**	**Study design**	**Population**	***N* number of KD patients**	**Age of KD patients (y)**	**Control**
	(28)	Population-based	Taiwan	2,748	1–18	Non-KD
**Allergic diseases**	**Asthma**	**Allergic rhinitis**	**Atopic dermatitis**	**Urticaria**	**Acute conjunctivitis**	
Adjusted odds ratio	0.89	1.44	1.22	1.82	1.09	
95% confidence interval	0.74–1.08	1.23–1.70	1.06–1.39	1.54–2.14	0.89–1.32	

*KD, Kawasaki disease; y, years*.

A previous study comprising 256 patients noted that wheezing was common among patients with KD with 12.5% of KD patients revealing wheezing at initial evaluation (29). In a study using linked population data, Webster et al. (12) found that admission for allergic diseases like asthma occurred more in KD patients than in controls. Furthermore, a population-based cohort study performed by Tsai et al. (14) that included patients under the age of 1 year reported that the rates of allergic diseases, including atopic dermatitis, asthma, and allergic rhinitis, were significantly higher in KD patients compared with the control group. As for individual allergic diseases, an increased risk of concomitant atopic dermatitis was found in male subjects with KD between 1 and 5 years of age (odds ratio, 3.02) in another population-based cohort study (15). However, in this study, the risk of atopic dermatitis was not increased in female KD children within the same age range. Some differences resulted from study design, including case-control or cohort studies, follow-up period, and selection of control group regarding atopic dermatitis in KD. In the cohort study, due to the consideration of age of onset, some atopic dermatitis had occurred before rather than after KD diagnosis.

Hwang et al. (15) further stratified KD patients by the presence of coronary lesions. These results revealed an increased risk for concomitant allergic diseases in male subjects without coronary dilatation (odds ratio, 1.74). This study also reported that any type of allergic disease was significantly higher in patients with KD aged between 1 and 5 years old (adjusted odds ratio, 3.02).

## Abnormal Type 2 Inflammation, Imbalance T Helper Type 17/Regulatory T Cell, and Other Immunopathogenesis in Kawasaki Disease

Some studies have suggested that children with KD and those with allergic disease may share a common biological background. Evidence has been found for both Th1 and Th2 cytokines activating the immune systems triggered by a KD etiological agent. Intense IL-1β, tumor necrosis factor α and IL-6 are also produced in acute KD patients (Figure 2) (22, 30, 31). IL-31 has also been reported to be involved in Th2-mediated diseases, such as allergic diseases, as well as KD (32). Eosinophils play an important role in type 2 inflammation (33). Previous studies have reported that patients with KD had higher eosinophils, Th2 cytokines IL-4, IL-5, and eosinophil cationic protein (ECP) levels than controls (17, 25). The eosinophil-related mediators IL-4, IL-5, and ECP increased significantly after IVIG treatment. Kuo and his colleagues demonstrated that eosinophil levels were highly elevated in the acute stage of KD both before and after IVIG treatment. High levels of post-IVIG eosinophil are represented in patients who respond well to IVIG (34). However, the causality in the association between eosinophilia and IVIG has been obscure. Lin et al. (35) identified that 38 patients with enterovirus infections treated with 1 g/kg IVIG had elevated eosinophil counts, as well as in 171 KD patients treated with 2 g/kg IVIG. The eosinophil percentage was shown to be significantly elevated in KD patients prior to IVIG treatment but was not found in enterovirus before IVIG treatment. Following IVIG treatment, elevated eosinophils were found both in KD and enterovirus, but a greater increase was observed in KD. Several factors, including IVIG brand, may influence eosinophils profile in KD (36). Eosinophils are considered potent multifunctional cells that play regulatory roles and have been reported to maintain homeostasis conditions by modulating IgA production in the intestine (37). IVIG administration-induced Th2 pathways with IL-4 involvement played a vital role in mediating inflammatory suppression by modulating Fc receptors in a mice model (38).

IgE was found to be especially elevated in the 2nd week of KD in spite of the lack of IgE in IVIG, suggesting an important role in IgE-mediated immunity in KD (17, 18, 39, 40). An -increase in IL-4 levels was found in serum from KD patients. In addition to type 2 inflammation phenotype stimulation, IL-4 has gained more attention as part of the key process of vascular injury by upregulating vascular cell adhesion molecule-1 in KD (41).

IL-4 is a general inducer of the low-affinity IgE receptor Fc epsilon RII/CD23. Fc epsilon RII demonstrated high absolute counts of CD23+ cells in the acute stage of KD on peripheral blood macrophages/monocytes and B lymphocytes in 12 and 10 patients with KD, respectively, using a fluorescence-activated cell sorter. It can be inferred that low-affinity IgE receptors are activated on peripheral blood macrophages/monocytes and B lymphocytes (39, 42). Multidimensional intracellular signaling pathways are triggered by the ligation of Fc epsilon RII (43). However, the absolute counts of CD23+ peripheral blood macrophages/monocytes in five KD patients with CAL were lower than those in 35 patients without CAL (44, 45). Circulating elevated levels of soluble CD23 in the serum of 33 KD patients during the acute stage were found compared to age-matched control subjects (46).

Connection of IgE to its high-affinity receptor Fc epsilon RI on basophils and mast cells plays an important role in the development of allergies (47). The engagement of Fc epsilon RI receptors combined with calcium/calcineurin signals induced the activation of the nuclear factor of activated T-cell (NFAT) proteins and also caused great concern in KD after processing the bioinformatics analysis with DAVID and the Kyoto Encyclopedia of Genes and Genomes (30).

Endogenous cysteinyl leukotriene E4 in the urine of 10 patients with KD increased significantly in KD compared to controls, suggesting that cysteinyl leukotrienes are involved in the pathophysiology of KD (48). *In vitro* stimulated polymorphonuclear cells from 19 KD patients obtained in the convalescent phase produced more Leukotriene B4 (LTB4), a chemo-attractant mediator and immunomodulator (49, 50). Other researchers reported increased serum-LTB4 concentration in both acute and convalescent phases. Another study reported that the plasma immunoreactive-leukotriene C4 level in patients with KD was significantly higher than that of healthy controls (29).

Essential vasculature-remodeling factor matrix metalloproteases-9 (MMP-9) contributes to extracellular remodeling in the airflow obstruction of asthma and the formation of coronary aneurysms in KD (51, 52). Epigenetic methylation status displayed an opposite tendency in MMP-9 transcriptional expression (52). The immunomodulatory T cell phenotype modulates allergic sensitization (53). A significant reduction in anti-inflammatory IL-10 levels in patients with asthma was identified when compared to control subjects, and IL-10 secretion was markedly lower in children with atopic dermatitis. Studies have also demonstrated that the expression levels of regulatory T cells' transcription factor (FoxP3) were significantly down-regulated in children with acute KD (Figure 2) (54). Previous reports have shown that regulatory T cells significantly increased after infliximab; FoxP3 transcriptional levels increased after IVIG (55, 56). Furthermore, children with acute KD were shown to have lower plasma transforming growth factor (TGF)-β concentrations (54).

The pro-inflammatory Th17 lymphocytes that produce IL-17 influence asthma inflammation and are associated with IgE production (57). Patients with KD had higher Th17 cytokines and transcription factors (Figure 2) (54, 56). Down-regulation of Th17 cells and cytokines was monitored following anti-inflammatory IVIG therapy (58).

The lungs' classically activated macrophages (M1) expressing proinflammatory cytokines and alternatively activated macrophages (M2) expressing Th2 cytokines both participate in the pathogenesis of asthma (59). One recent study suggested that M1 and M2 cells are distinct cell subtypes that play separate roles in the immunopathogenesis of KD and also showed M2 to be predominant (Figure 2) (60).

## Limitations

This study and the documents used had certain limitations. We have found that Taiwan's health insurance database has some repeated or different calculation methods, while Japan, Europe, and the United States lack relevant research. Some population-based studies in Taiwan's health insurance database also fail to show the sequence of KD and allergic diseases due to the study's design. Since KD primarily occurs in children younger than 5 years old, and many allergies occur after the age of 5, the study of KD after allergic diseases did not include older allergic children. Furthermore, allergies later classified as a non-allergic group did not seem appropriate (28).

## Summary

More consistent conclusions from relevant research can be drawn about the increased risk of allergic rhinitis in patients with KD (Table 2; Figure 1). This correlation may potentially come from the increase of IL-5, eosinophils, and total IgE in children with KD. Whether other allergic diseases (food allergy, conjunctivitis, urticaria, etc.) also increase after KD warrants additional study. Future statistical calculations and analysis of inconsistent data on asthma and atopic dermatitis in KD patients will advance the care of KD (Tables 1, 3). Therefore, considering these immunologic factors as contributing to the allergic diseases observed after KD is important. More attention should be paid to the appearance of allergic diseases during and after the course of KD. In some of the aforementioned studies, more allergic diseases were observed in KD subjects. Regarding the effect of CAL on allergic diseases, evidence from previous studies has been inconclusive. Chronic allergic diseases, especially allergic rhinitis and urticaria, have been shown to be associated with KD during childhood. KD may have long-term public health implications, so prevention strategies ought to be implemented. However, long-term outcomes of pediatric KD occurring during early childhood after the age of 20 years old remains unknown. Future prospective studies are needed to determine the long-term effects on allergic diseases among children with KD after the age of 20 years old. Allergic symptoms and even an allergen survey are suggested as a component in KD follow-up visits.

## Author Contributions

P-YH, Y-HH, MG, H-CK, and L-SC drafted the article, carried out conception, design, and initial analyses, and approved the final manuscript as submitted. All authors contributed to the article and approved the submitted version.

## Conflict of Interest

The authors declare that the research was conducted in the absence of any commercial or financial relationships that could be construed as a potential conflict of interest.

## Abbreviations

CAL: coronary artery lesions
ECP: eosinophil cationic protein
Ig: immunoglobulin
IL: interleukin
IVIG: intravenous immunoglobulin
KD: Kawasaki disease
LTB4: Leukotriene B4
M1: classically activated macrophages
M2: alternatively activated macrophages
MMP-9: matrix metalloproteases-9
TGF: transforming growth factor
Th: T-helper.
